# Unlocking the Mystery of Patella Dislocation—Diagnostic Methods in Pediatric Populations: A Comprehensive Narrative Review

**DOI:** 10.3390/jcm14041376

**Published:** 2025-02-19

**Authors:** Ewa Tramś, Ignacy Tołwiński, Marcin Tyrakowski, Dariusz Grzelecki, Jacek Kowalczewski, Rafał Kamiński

**Affiliations:** 1 Department of Musculoskeletal Trauma and Orthopaedics, Centre of Postgraduate Medical Education, Gruca Orthopaedic and Trauma Teaching Hospital, Konarskiego 13, 05-400 Otwock, Poland; 2 Department of Orthopedics and Rheumoorthopedics, Centre of Postgraduate Medical Education, Professor Adam Gruca Orthopedic and Trauma Teaching Hospital, Konarskiego 13, 05-400 Otwock, Poland; marcintyrak@gmail.com (M.T.); dariuszgrzelecki@gmail.com (D.G.);

**Keywords:** knee instability, patellofemoral, knee diagnostic, pediatric

## Abstract

**Background/Objectives:** The diagnostic guidelines for pediatric patellofemoral instability (PFI) remain incomplete. PFI remains a challenging issue as it affects the biomechanics of the knee joint, triggers anterior knee pain, and is linked to the development of early-onset osteoarthritis. The diagnostic process is complicated by numerous anatomical factors that must be considered. This review aims to consolidate current knowledge presented in the literature on radiological diagnostics for PFI in pediatric populations, with the application of all imaging techniques—including ultrasonography (US), magnetic resonance imaging (MRI), computed tomography (CT), and radiography (RTG)—which enable the evaluation of anatomical risk factors critical for the diagnosis, prevention, and treatment of PFI. **Methods**: A search of the PubMed/MEDLINE database was conducted to identify relevant studies from 1975 to 2024. The search terms were as follows: (patellar or patella) and (instability or displacement or dislocation) and (diagnostic or diagnosis or imaging or radiographic). A total of 2743 articles were retrieved, which were screened to yield 29 studies for further review. These studies were then divided into seven categories regarding the diagnostic methods: risk factors, tibial tubercle trochlear groove (TT-TG)/tibial tubercle posterior cruciate ligament (TT-PCL), MPFL injury and cartilage damage, patella and trochlear dysplasia, torsional abnormalities, coronal plane alignment, and genetics. **Results**: The methods presented statistically significant differences, with those most commonly used for the diagnosis of patella dislocation being TT-TG index, MPFL rapture, and trochlear dysplasia. **Conclusions**: In summary, multiple diagnostic tools, including MRI, CT, X-ray, and physical examination, are available for the assessment of PFI, each contributing to treatment decisions. Although MRI remains the primary diagnostic tool, further research is needed to establish more precise diagnostic criteria.

## 1. Introduction

Patellofemoral instability (PFI) is a prevalent issue among knee injuries, especially in the pediatric population, with an incidence of approximately 50 cases per 100,000 individuals. The likelihood of onset is notably higher in females aged 10 to 17, peaking at age 15, and is often associated with a positive family history [1,2,3]. PFI arises from the incongruence between the patella and the femoral trochlear groove, encompassing a spectrum of disorders from mild maltracking to lateral patellar dislocation. Over 80% of primary patellar dislocations are attributed to a non-specific, non-contact trauma mechanism [4]. The recurrence rates for patellar dislocations—which contribute to PFI—are significantly high, ranging from 30 to 40% after non-operative treatment of a first-time dislocation to 70 to 80% in patients presenting with certain risk factors [5,6]. Injury to the medial patellofemoral ligament (MPFL) is highly prevalent, with an incidence approaching 100% following an initial patellar dislocation. However, a multitude of anatomical factors, such as trochlear dysplasia or skeletal immaturity [5,6,7] and alignment abnormalities (including an abnormal Q angle or excessive lateral displacement of the tibial tubercle or patella alta [1,2]), also contribute to the development of PFI. Furthermore, the risk of progressive cartilage damage and osteoarthritis (OA) after lateral patellar dislocation increases sixfold after the first dislocation, which leaves many young patients vulnerable to developing OA in adulthood [3]. This review aims to critically evaluate the most precise methods for diagnosing PFI in pediatric patients, utilizing advanced diagnostic tools informed by established risk factors. This approach is expected to facilitate more accurate and informed decision-making in the treatment of this condition.

## 2. Materials and Methods

**Figure 1:**
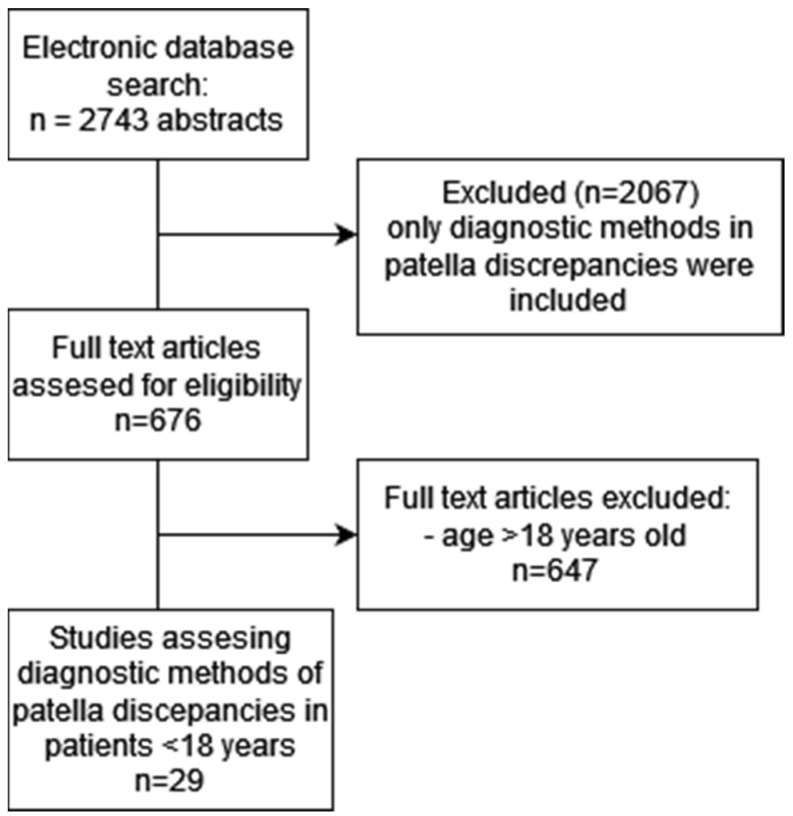
Flowchart of the article selection process.

A comprehensive search of the PubMed/MEDLINE database was conducted to identify relevant studies published between 1975 and 2024. The search was performed between May and June 2024 using the search terms (patellar or patella) and (instability or displacement of dislocation) and (diagnostic or diagnosis for imaging or radiographic). A total of 2743 articles were retrieved. Selected articles were limited to those written in English and specifically focused on identifying and diagnosing patellar discrepancies. Articles primarily addressing the treatment of patellar instability, with only a brief mention of diagnostic aspects, were excluded. Specified inclusion and exclusion criteria are detailed in Table 1. Two authors independently screened the titles and abstracts to assess their relevance. Any discrepancies between reviewers were resolved through discussion. After this stage, 678 articles were identified as potentially relevant. The selected articles underwent a thorough full-text review to ensure they met the eligibility criteria. After further eliminating all articles that were not prospective or retrospective studies, clinical trials, and cases concerning patellofemoral disorders in patients over 18 years, 29 articles were finally selected and included in this review. The final studies were systematically analyzed, and relevant data were extracted. A narrative synthesis of the included articles was conducted. Key findings were summarized in a comparative table (Table 2) to facilitate clear interpretation of diagnostic methodologies. A PRISMA flow diagram (Figure 1) was used to illustrate the selection process, providing a visual summary of the study identification, screening, eligibility assessment, and final inclusion.

## 3. Results

The 29 included studies were divided into seven categories regarding the type of diagnostics used: risk factors, tibial tubercle trochlear groove (TT-TG)/tibial tubercle posterior cruciate ligament (TT-PCL), MPFL injury and cartilage damage, patella and trochlear dysplasia, torsional abnormalities, coronal plane alignment, and genetics. The studies and their results are listed in Table 2.

**Table 2:** Synthesis of the included studies.

Reference	Study Design	Data Collection	Age	Study Aim	Diagnostic	Relevant Factors	Patella Dislocation
Askenberger 2016 [8]	Prospective	103 patients vs. 69 controls	9–14 years	Determining patellofemoral morphology and PFI risk factors	MRI (1.5-T; T-1-weighted)	Trochlear dysplasia, trochlear depth, sulcus angle (SA), lateral patellar tilt, TT-TG, patella alta (Caton Deschamps CD)	First-time
Balcarek 2010 [9]	Retrospective	22 patients vs. 21 controls group (adults)	11–15 years	Comparing MPFL injury in pediatric population with adults and evaluating trochlear groove anatomy in growing knee	MRI (1.5-T/3-T; T1-weighted)	MPFL, trochlear dysplasia: SA, trochlear depth, trochlear asymmetry	First-time
Bayhan [10]	Retrospective	77 vs. 792 controls	5–15 years	Assessing the variations in TT-TG distance and angle as a function of age and gender	MRI	TT-TG distance and TT-TG angle	Patella instability
Choi 2021 [11]	Retrospective	852 knees (663 patients)	4–18 years	Analyzing age and gender variations in the patellofemoral joint	1.5 T MRI T1	TT-TG, SA, percent tibial tuberosity location, Trochlear Facet asymmetry (TFA), femoral depth, lateral trochlear inclination, alignment	
Clifton 2017 [12]	Retrospective	28 patients vs. 484 controls	0–16 years	Confirming reliability and reproducibility of TT-PCL in pediatric population	MRI (1.5-T/3-T)	TT-PCL	
Dickens 2014 [13]	Retrospective	76 patients vs. 495 controls	9 months to 16 years	Determining TT-TG distance in pediatric population and confirming that MRI measurements are reliable and reproducible	MRI (1.5-T/3-T; T2-weighted)	TT-TG	Recurrent
Duppe [14]	Retrospective	66 patients vs. 132 controls	8–18 years	Delineation of differences in morphological parameters between children with patella instability and control	1.5 T MRI, T1, T2	Medial femoral condyle height, width, MPFL, Insall–Salvati (IS), CD, Fulkerson angle, inclination angle, SA, sulcus depth, TT-TG	
Igrek [15]	Retrospective	306	Adolescent	Investigating risk factors for osteochondral fractures	MRI	Cartilage (osteochondral fracture), Beighton, patellar high, lateralization, trochlear morphology, patella types, TT-TG, TT-PCL, lateral patellar displacement	Acute first-time
Kang [16]	Prospective	53 and 53 controls	5–22 years	Evaluating the patellar cartilage in patients with PFI	MRI T2	Cartilage damage (mean T2 relaxation time)	
Kim 2015 [17]	Retrospective	53 patients vs. 53 controls	8–21 years	Comparing MRI with and without PFI and evaluating severity of cartilage damaged	MRI (1.5-T; T-2-weighted)	Trochlear dysplasia: trochlear inclination angle, TFA, trochlear depth, Dejour classification; injury patterns; patellofemoral alignment: patellar heigh, TT-TG, lateral patellofemoral angle	
Park [18]	Retrospective	698 knees	4–18 years	Evaluating the reliability of the TT-TG distance and TT-TG index in pediatric population	3 T, T2 MRI	TT-TG distance, TT-TG index	
Park [19]	Retrospective?	87 vs. 509 controls	>18 years	Analyzing TT-TG and TT-PCL distance in children with no PFI and proposing cut-off values for prediction of increased risk of PF instability	MRI	TT-TG and TT-PCL distance	Patella instability
Seeley 2012 [20]	Retrospective	111 patients	11–18 years	Characterizing the patterns of MPFL, VMO and osteochondral injury; evaluating risk factors of recurrence	MRI (1.5T; T1/T2-weighted)	MPFL, vastus medials oblique (VMO), IS, trochlear dysplasia, TT-TG, IS, SA, trochlear depth, Wiberg classification, Dejour classification, lateral trochlear inclination	Acute first-time
Schranz 2021 [21]	Retrospective	30	12–18 years	Determining the relationship between static femoral anteversion and internally rotated gait	MRI 1.5T, T1; 3D gait analysis	3D gait analysis, femoral anteversion	PFI recurrent
Yeoh [22]	Retrospective	43	10–17 years	Comparing TT-TG index and distance between one-time and recurrent patella dislocation	MRI	TT-TG index, TT-TG distance	One-time and recurrent patella dislocation
Yilmaz 2016 [23]	Retrospective	20 patients vs. 20 controls	10–16 years	Determining the morphological features of the patella and patellar tendon that may predispose to acute first-time dislocation	MRI (1.5-T, T1/T2-weighted)	SA, trochlear sulcus depth, IS, CD, patellar width, patella width, length	Acute first-time
Yilmaz [24]	Retrospective	40 adolescents	10–16 years	Evaluating differences in the measurement techniques between radiology specialists and orthopedic surgeons	1,5 T MRI (white)	IS, BP (Blackburne-Peel), CD, modified IS	
Zheng [25]	Prospective	123	<18 years	Investigating the injury characteristics of MPFL and analyzing correlations between MPFL and cartilage injury	MRI (T2)	MPFL, cartilage (chondral and osteochondral damage)	Acute first-time lateral patella dislocation
Zheng [26]	Prospective	32 m vs. 57 f	12–18	Exploring gender differences in articular injuries	MRI 1,5T	MPF, cartilage lesions	Acute dislocation
Bernholt [27]	Prospective	57	10–18 years	Evaluating the utility of TT-TG measurement on Merchant radiographs compared to MRI	Merchant X-ray, MRI	TT-TG	
Lewallen 2013 [5]	Retrospective	210 patients	9–18 years	Determining predictors of recurrent instability and describing demographics of patients with first-time patella dislocation	X-ray lateral	Dejour classification: Crossing sign Supratrochlear spur Double contour; CD and IS	First-time and recurrent
Lippacher 2011 [28]	Retrospective	20 patients	6–14 years	Evaluating true lateral radiographs of typical radiological findings in patients with open growth plates	X-ray true lateral	Crossing sign Supratrochlear spur Double contour	Recurrent
Stepanovich 2016 [29]	Retrospective	36 vs. 27 controls	Patients with open physis	Examining several measurements of trochlear dysplasia and assessing which best correlates with patellar instability	X-ray (AP, 30 lateral, merchant) and MRI (1.5-T, T1- weighted)	Dejour classification; trochlear depth, lateral trochlear inclination, medial condyle trochlear offset, TT-TG	Recurrent
Simon 2014 [30]	Prospective	158 lower limbs	4.5–25 years	Determining patellar orientation in the transverse plane during observational gait analysis	Physical examination, 3d gait analysis	Femoral anteversion, 3D gait analysis	Spastic dysplegic
Malecki [31]	Prospective	50 vs 199 control	10–17 years		Genetics	COL Brighton score	
Huang [32]		15 habitual, 18 recurrent	9–15	Comparing the radiological features of recurrent and habitual patella dislocation	Hip/knee/ankle CT	Trochlear depth index, lateral trochlear inclination, SA, TT-TG, TT-TG ratio, TT-TG angle, femoral anteversion angle, tibial external rotation angle, Dejour and Wiberg classification, IS, CD	
DeVries	Prospective	203 patients		Diagnosis of trochlear dysplasia using US and its correlation with patellar Instability	US	Trochlear dysplasia, SA, Trochlear depth	
Nietosvaara	Prospective	33 patients vs. 25 controls		Assessment of SA via US and its influence on prevalence of patellar dislocation	US	SA, Trochlear dysplasia	
Øye	Prospective	174 newborns (82 girls and 92 boys)		Use of ultrasonography to define the normal anatomy and natural variations in the femoral trochlea in a newborn population	US	SA, Trochlear Index	

### 3.1. Diagnostic Methods

MRI is the most commonly used method for diagnosing patellar dislocation in the pediatric population. A total of 19 studies evaluated MRI exclusively, while 2 studies compared MRI with X-ray. MRI appears to be the diagnostic method of choice due to its ability to evaluate osteochondral injuries, loose bodies, soft tissue damage, and to identify risk factors for patellar instability. MRI showed 85% sensitivity in the diagnosis of MPFL injury [33,34,35].

There were two additional studies using X-ray for diagnosis. However, radiographs should be performed after first-time traumatic patella dislocation as well as before surgery planning, including AP weight-bearing and lateral and Merchant views (femoropatellar axial view). In this way, the physician can detect osteochondral fractures and dislocation of the patella, as well as diagnose patella dislocation risk factors. True lateral radiographs are used for assessment of patellar higha according to the measured length of the patella over the length of the patellar tendon (IS), or the length of the patella articular surface over the distance from the patella to the tibial plateau (BP). The Dejour classification encompasses three risk factors (crossing sign, supratrochlear spur, double contour), also classified based on true lateral radiographs. Additional standing alignment radiographs help to detect other skeletal abnormalities such as genu valgum [36,37,38].

No studies were focused on the diagnostic evaluation of patellar dislocation in children using CT. Only one study included CT in the diagnostic process for patellar dislocation. This limited use may be attributed to the radiation exposure associated with CT, compared to the radiation-free nature of MRI. However, CT offers greater detail in assessing osseous structures, including measurements such as the TT-TG distance, SA, and patellar tilt. Additionally, CT allows for the identification of rotational abnormalities. [35,38].

Ultrasonography is shown in the literature as a potential addition to the diagnosis and management of patellar dislocation. Given its rapid and non-invasive nature, it serves as an efficient screening tool, helping clinicians in determining the necessity for further diagnostic evaluation and therapeutic intervention. Notably, two studies included in our review demonstrate the use of ultrasonography in assessing trochlear dysplasia through the measurement of sulcus angle (SA) and trochlear depth, highlighting its potential role in the diagnostic approach to patellofemoral instability [39,40]. A comparison of these imaging techniques is presented in Table 3.

### 3.2. Risk Factors

Various risk factors have been suggested for adult populations. Identifying risk factors in pediatric populations with immature growth plates is a challenging but important diagnostic path for better injury prevention. Askenberger et al. characterized patellofemoral morphology in children with primary lateral patellar dislocation on MRI and evaluated anatomic patellar instability risk factors (APIF). They found that SA, trochlear depth, TFA, lateral trochlear inclination, lateral patellar displacement, patella tilt, patellar length, patellar tendon length, I-S, C-D, and TT-TG distance were significantly different between the study and control groups (*p* < 0.05). In particular, 79% of patients had 2–4 APIF in the study group, compared to 7% of controls. Interestingly, the most common instability factor in the study group was trochlear dysplasia (74%), while TT-TG distance did not exist as an isolated divergence [8]. Duppe compared the MRIs of children with patella dislocation with those from a control group. They found statistically significant differences in I-S, C-D, Fulkerson angle, patellar inclination, SA, medial and lateral portion, sulcus depth, external trochlear facet/internal trochlear face, TT-TG, trochlear groove, and medial and lateral angle % of SA. They also evaluated age-dependent parameters defined by age group and found that SA-bone, SA-cartilage, sulcus depth-bone, and lateral condyle-cartilage were significant. The authors suggested that physicians should consider the age of patients when evaluating patellar instability [14]. Choi et al. also analyzed age and gender variations associated with the patellofemoral joint. Significant differences (*p* < 0.05) were found in TT-TG, SA, percent tibial tuberosity location, TFA, and femoral depth (FD). The authors suggested that morphological changes in the patellofemoral joint may facilitate the diagnosis of patellofemoral joint pathologies [11]. Kim et al. compared sex and age-mashed groups with and without PFI. They found significant differences (*p* < 0.0001) in MR measurements of trochlear dysplasia and patellofemoral alignment when comparing the PFI and control groups. A significant correlation was also found between MPFL injury and the degree of patellar cartilage damage in the medial area (*p* < 0.05); however, there was no correlation between trochlear dysplasia and patella cartilage damage, and only patella alta was correlated with cartilage injury [17]. Lewallen et al. analyzed radiographs performed within 4 weeks of the initial injury. Trochlear dysplasia was determined by Dejour classification and assessed according to two indices: CD and IS. Of the patients, 48 (37.8%) had trochlear dysplasia and 100 (45%) had patella alta. A total of 24 knees were treated operatively within a mean time of 30 days, 8 of which presented recurrent instability. Of the 198 remaining cases, 76 (38.4%) had recurrent subluxation or dislocation with a mean follow-up of 3 years. Recurrent instability was strongly (2.5 times more likely) associated with trochlear dysplasia (hazard ratio 2.57; *p* < 0.01). However, patella alta did not appear to be a risk factor. They also found that patients involved in sport at the time of initial dislocation and those with immature physes had increased hazard ratios (1.69 and 1.58), and were nearly 70% and 60% more likely to experience recurrent events. In conclusion, 69% of skeletally immature patients had failed non-operative treatment for trochlear dysplasia and were 3.3 times more likely to have recurrent instability [5].

Across the studies, there is a consensus emphasizing the significance of trochlear dysplasia, patella alta, and TT-TG distance as key risk factors, particularly in younger, skeletally immature populations. Age and activity level should be carefully considered when evaluating these risk factors to enhance the diagnostic accuracy and optimize treatment outcomes.

### 3.3. MPFL Injury and Cartilage Damage

Some studies have associated cartilage injury with MPFL lesions. Kang et al. investigated cartilage injury and compared adolescents with and without PFI in relation to patellar cartilage damage. T2 mapping was obtained for the maximal, lateral, and central zones of the patella. In the patella instability group, 98.1% of patients had articular cartilage lesions in at least one location. Lesions occurred most frequently in the medial facet (seven patients). The mean T2 relaxation times were statistically significant in all six locations in patella when comparing PFI and control groups (*p* < 0.001). They concluded that T2 relaxation time positively correlates with morphological grades of cartilage lesions [16]. Furthermore, Seeley et al. have evaluated osteochondral and MPFL injuries, as well as risk factors for recurrence, through analyzing MRIs from first-time patella dislocation patients. Of these patients, 78.4% had identified MPFL injury and 56% presented with edema of VMO. Osteochondral injuries occurred in 34% of patients, of which 66% presented injury on the medial facet of the patella. Patients with patellar and femoral injuries were significantly more likely to have recurrence. In patients with injury of MPFL, 67% had trochlear dysplasia, with Dejour dysplasia type B (28%) being the most frequent. TFA, TT-TG, SA, and trochlear depth presented significant differences (*p* > 0.05) when compared to the no-injury group. Recurrence occurred in 30.6% of patients; however, in the younger population (11–13 years), this figure was 43.5%. Recurrence was not correlated with MPFL or VMO strain [20]. Another study compared MPFL and cartilage injury between a pediatric study group and an adult population. MPFL injury was found in 90.9% of the pediatric population and 100% of the adult population, where 27% of children and 33% of adults showed cartilage lesions. Differences between the groups in regard to parameters of trochlear dysplasia (SA, trochlear depth, trochlear asymmetry) were not significant [9]. Likewise, Zheng et al. have analyzed the correlation between MPFL injury and cartilage lesions. The most common MPFL injury locations were isolated patellar insertion (47 cases), isolated femoral attachment (41 cases), and multiple injury sites (31 cases). Significantly higher rates of lesions were found in cases with complete MPFL tears (33 out of 69) when compared to partial tears (18 out of 54) [25]. In a separate study, Zheng et al. investigated gender-related differences in articular injuries. They found that males exhibited significantly more frequent complete MPFL tears (50%) compared to females (29.8%; *p* = 0.049), as well as a higher incidence of articular cartilage lesions of the patella (40% in males vs. 21.1% in females; *p* = 0.043) [26]. A multicenter retrospective study analyzed cartilage injuries according to the mechanisms of showing cartilage lesions, including sports, simple falls, and daily activities. A significant difference in the occurrence of osteochondral fractures was found in the sports injury group (*p* = 0.001) and in patients with hypermobility (*p* = 0.041). Additionally, in the presence of osteochondral fractures, significantly higher measurements were observed for TT-TG, TT-PCL, and lateral patella displacement (*p* = 0.001) [15]. MPFL injuries are often associated with cartilage damage and recurrent instability, particularly in younger individuals and males. Prompt imaging and diagnosis are essential for effective management and prevention.

### 3.4. Patella Alta and Trochlear Dysplasia

Yilmaz et al. compared 20 children with acute first-time patella dislocation to 20 patients in a control group. The study group exhibited a significantly greater SA (*p* = 0.001), significantly reduced sulcus depth (*p* = 0.001), smaller patella width (*p* = 0.002), shorter median patella length (*p* = 0.015), and reduced patellar volume (*p* = 0.022) when compared to the control group. However, there were no significant differences in patellar height (*p* > 0.05) or patellar tendon length (*p* > 0.05). Patella alta (as measured according to the I-S ratio) was found in 16 patients (80%) in the study group, compared to 3 patients (15%) in the control group [23]. In another study, the same authors evaluated patella alta measurements conducted by three orthopedic surgeons and three radiology specialists. They found that radiology specialists demonstrated a higher level of interobserver agreement (as measured by Fleiss’ Kappa) across all measurement methods [24]. Lippacher et al. evaluated true lateral radiographs in children and adolescents with open growth plates and recurrent patella dislocation. They retrospectively analyzed lateral X-rays in 20 skeletally immature patients with trochlear dysplasia confirmed via MRI. The study found that each case exhibited at least one typical radiological sign: crossing sign (19 patients), supratrochlear spur or bump (6 patients), and double contour sign (15 patients). They concluded that true lateral radiographs are sufficient for diagnosing trochlear dysplasia in children [28]. Furthermore, Stepanovich et al. investigated both imaging methods for the diagnosis of patellar instability. Based on radiographic measurements, no patients in the control group were diagnosed with trochlear dysplasia. In contrast, within the patellar instability group, 1 patient had no dysplasia and 14 had dysplasia. However, only 25 of the 63 patients could be assessed using the radiographic Dejour classification. Using MRI-based Dejour classification on all 63 patients, 10 of the 27 control group patients exhibited type A dysplasia; meanwhile, in the patellar instability group, only 2 patients had no dysplasia, while 34 had dysplasia. The MRI Dejour classification had the lowest inter- and intra-observer reliability (k = 0.687 and 0.596, respectively), while all other measurements had values greater than 0.80. Additionally, MRI measurements including medial condyle trochlear offset (MCTO), trochlear depth index (TDI), lateral trochlear inclination (LTI), and TT-TG differed significantly between the experimental and control groups (*p* < 0.001), with TT-TG being directly correlated with the severity of trochlear dysplasia [29].

Moreover, ultrasonography (US) has been highlighted in the literature as a diagnostic tool in the management of patients with PFI. A prospective study by DeVries, Clarabelle A. et al. aimed to determine the prevalence of trochlear dysplasia and its association with a history of PFI or pain, using US in skeletally mature patients. High-grade trochlear dysplasia was defined as measurements in the 95th percentile or higher, indicated by an SA of ≥154° or a trochlear depth of ≤3 mm for females and ≤4 mm for males. The findings showed that the prevalence of high-grade trochlear dysplasia was 5.4% when assessed according to the SA and 9.9% when assessed via trochlear depth. Notably, knees with high-grade trochlear dysplasia, as determined by the SA, were 11 times more likely to have a history of patellar instability (*p* = 0.013). In conclusion, the study highlighted that approximately 10% of the general population exhibits high-grade trochlear dysplasia and a significant association with patellar instability was suggested [39].

Additionally, the work of Nietosvaara, Y. and K. Aalto highlights the role of ultrasonography in diagnosing patellar dislocation in the pediatric population. They observed that in knees with patellar dislocation, the cartilaginous SA ranged from 154 to 195 degrees, thus exceeding the normal range of 134–153 degrees. In summary, their research supports the use of the SA measured via ultrasonography as a potential diagnostic tool for patellar instability [40]. In another study, Huang et al. compared habitual and recurrent patellar dislocations in children using CT. They assessed trochlear dysplasia, tibial tubercle lateralization, and rotational deformity through hip/knee/ankle CT scans. They found a significant difference in the prevalence of Dejour dysplasia type C, which was observed in 57% of habitual patellar dislocations versus 4.5% in recurrent patellar dislocations. Additionally, habitual dislocations showed a higher prevalence of dysplastic patellae, with 66.7% classified as Wiberg type 3 compared to 9.1% in recurrent dislocations. Conversely, recurrent patellar dislocations presented with higher IS ratios (1.3 vs. 1.1; *p* = 0.034) and TERA values (38.4 vs. 31.3; *p* = 0.009) [32].

Ultrasonography (US) also appears to be a potentially valuable tool for predicting PFI in newborns. A study by Øye et al. has identified the SA and the Trochlear Index (TI) as the most reliable and reproducible parameters for assessing trochlear morphology. The overall mean SA was found to be 148° (SD 5.6). An angle exceeding 159° was classified as dysplastic, with 17 knees falling into this category. The overall mean TI was 2.21 (SD 0.05), and values below 2.11 were considered dysplastic, encompassing 11 newborns [47]. Structural abnormalities such as trochlear dysplasia and patella alta are major contributors to patellar instability. Comprehensive imaging, including ultrasonography, plays a crucial role in facilitating early diagnosis and guiding effective treatment strategies.

### 3.5. TT-TG/TT-PCL

The TT-TG distance is a useful measurement for planning surgical management in the pediatric population. Several studies have compared this parameter within pediatric populations or proposed the TT-PCL distance as a potentially more reliable and stable alternative. Dickens et al. have evaluated the TT-TG distance in a pediatric population, and confirmed that MRI measurements in children are both reliable and reproducible. The median TT-TG distance in their study group (patients with recurrent patellar instability) was significantly higher at 12.1 mm compared to 8.5 mm in the control group (*p* < 0.001). The study also showed a logarithmic progression of TT-TG distance relative to age. Intra-observer agreement for TT-TG measurements was excellent (weighted kappa, 0.966; 95% CI, 0.96 to 0.97; *p* < 0.001), as was the inter-observer agreement (weighted kappa, 0.957; 95% CI, 0.95 to 0.96; *p* < 0.001). Interestingly, 1.5T MRI measurements (9.3 mm) were significantly greater than those from 3T MRI (7.7 mm; *p* < 0.001) [13]. Park et al. compared the TT-TG distance with the TT-TG index and found that, while the TT-TG distance increased with age, the TT-TG index remained relatively constant. They suggest that the TT-TG index may be a more reliable measurement for patellar height in pediatric populations [18]. Another retrospective study confirmed that TT-TG distance (17.2 mm) and TT-TG angle (20.8°) were significantly higher in the patellar instability group compared to the control group (10.4 mm and 12.5°, respectively; *p* = 0.001). They also found a positive correlation between age and TT-TG distance (r = 0.243, *p* < 0.001), with the female population presenting a higher TT-TG angle compared to males (13.3° vs. 11.9°, *p* < 0.001) [10]. Yeoh et al. investigated the TT-TG distance and TT-TG index in pediatric patients, finding that those with recurrent patellar dislocation had a higher TT-TG distance (*p* = 0.026) and TT-TG index (*p* = 0.008) than those with a one-time dislocation. The risk of recurrence was notably higher when the TT-TG distance exceeded 14 mm (relative risk = 6.4) and when the TT-TG index was greater than 0.26 (RR = 6.7) [22]. Conversely, Bernholt et al. compared TT-TG measurements on Merchant view radiographs with TT-TG on MRI, finding a moderate correlation (Pearson correlation coefficient = 0.58; *p* < 0.001) between the two methods. They also confirmed that TT-TG was increased in the pediatric population with patellar instability, when compared to the control group. TT-TG measurements on radiographs (8.4 ± 7.7 mm) were, on average, 4.5 mm less than those on MRI (12.8 ± 4.4 mm; *p* < 0.001) [27].

Clifton et al. compared the TT-PCL distance in a pediatric population using MRI. They found a significant difference (*p* < 0.001) between measurements taken with 1.5 T and 3.0 T MRI scanners. The average TT-PCL distance was 19.0 mm on the 1.5 T magnet and 21.3 mm on the 3.0 T magnet. Intra-observer agreement was excellent (weighted kappa, 0.93; 95% confidence interval [CI], 0.92–0.94; *p* < 0.001), and interobserver agreement was high (weighted kappa, 0.80; 95% CI, 0.78–0.83; *p* < 0.001). However, there was no significant difference in TT-PCL distance between patients with and without patellar instability (*p* = 0.07). A significant difference was observed between sex subgroups; namely, TT-PCL distance was significantly greater in males compared to females (*p* < 0.05). Consistent with findings in previously described studies, the TT-PCL distance significantly increased with age [12]. On the other hand, Park et al. found that the median TT-TG and TT-PCL distances were significantly larger in the instability group (16.1 mm and 24.41 mm), respectively, when compared to the control group (8.18 mm and 19.48 mm). They also proposed cutoff values of 23.68 mm for TT-PCL distance, with 63.9% sensitivity and 65.3% specificity, and for a TT-TG distance of 14.9 mm, with 66% sensitivity and 81.9% specificity [19]. Across the studies, there is a consensus supporting the use of TT-TG and TT-PCL as reliable metrics for evaluating patellar instability, both demonstrating strong interobserver reliability. These parameters underscore the importance of considering patient age and MRI settings to ensure accurate measurements. TT-TG and TT-PCL are dependable indicators for assessing patellar instability and risk of recurrence, with significant implications for surgical planning.

### 3.6. Torsional Abnormalities

Schranz et al. analyzed 30 adolescents with PFI using 3D gait analysis and MRI-based torsional profiling. The obtained Pearson correlation coefficients showed no significant association between femoral anteversion or tibial torsion and parameters such as hip rotation in stance, hip rotation in swing, pelvic range of motion, pelvic rotation, or foot progression angle in stance. The authors recommended the inclusion of gait analysis in the preoperative assessment of adolescents, as static parameters alone may be insufficient [21]. Simon et al. examined 158 lower limbs in children with spastic diplegia, measuring femoral anteversion (FA) through physical examination using Netter’s method. They set a cutoff for FA at >30 degrees. Additionally, they performed kinematic 3D-computerized gait analysis to investigate the causes of an inward-facing patellar gait. Among the children, 71% exhibited excessive FA, with 56% displaying excessive internal hip rotation associated with internal pelvic rotation in 18% of cases. Another 44% showed excessive internal pelvic rotation with external hip rotation at 18%. In 42%, the hip and pelvis rotated in opposite directions. In the 25% without excessive FA, 61% had isolated internal pelvic rotation, 30% had isolated internal hip rotation, and 9% had both [30]. Torsional abnormalities play a significant role in patellar instability but require dynamic assessment, such as 3D gait analysis, for accurate diagnosis and effective management.

### 3.7. Genetics

Ligamentous laxity is associated with various musculoskeletal injuries, and joint hypermobility is a known risk factor for patellar dislocation. Hypermobility results from collagen abnormalities, as a primary component of the MPFL, which serves as the main stabilizer of the patella. In their study, Malecki et al. investigated the coexistence of polymorphisms in the COL1A1 and COL5A1 genes among adolescents with recurrent patellar dislocation. Joint laxity was defined as a Brighton score > 4. PCR-RFLP analysis was used to determine the genotype distribution for selected single nucleotide polymorphisms. While no significant differences were found between groups concerning the analyzed genes, the Brighton score was significantly higher in the recurrent patellar dislocation group compared to the control (*p* < 0.001), with notable differences between sexes (*p* = 0.004 for females and *p* = 0.031 for males). In conclusion, there is a strong need to further investigate the genetic and molecular causes of joint laxity in relation to patellar instability [31].

#### Results Summary

The findings of this comprehensive review highlight the multi-faceted nature of patellofemoral instability (PFI) diagnosis in the pediatric population. An analysis of 29 studies identified seven diagnostic categories: risk factors, TT-TG/TT-PCL measurements, MPFL injuries, patellar and trochlear dysplasia, torsional abnormalities, coronal plane alignment, and genetics. Table 2 summarizes the frequency and significance of key patellofemoral morphology risk factors identified across the studies considered. Among these, trochlear dysplasia and TT-TG distance emerged as the most frequently mentioned and statistically significant risk factors, particularly in the context of recurrent dislocations.

MRI was identified as the diagnostic method of choice, demonstrating a high sensitivity (85%) for the detection of MPFL injuries while also providing the capability to assess multiple risk factors, including trochlear dysplasia, patellar height, and TT-TG distance. While X-rays remain a viable first-line diagnostic tool, particularly for evaluating patellar height and Dejour classification, their reliability is often limited by the need for precise imaging angles. Similarly, CT scans, which offer superior visualization of osseous structures, are infrequently used in pediatric populations due to concerns about radiation exposure. Ultrasonography, although less commonly employed, has shown promise as a radiation-free alternative for evaluating the sulcus angle and trochlear dysplasia.

**Figure 2:**
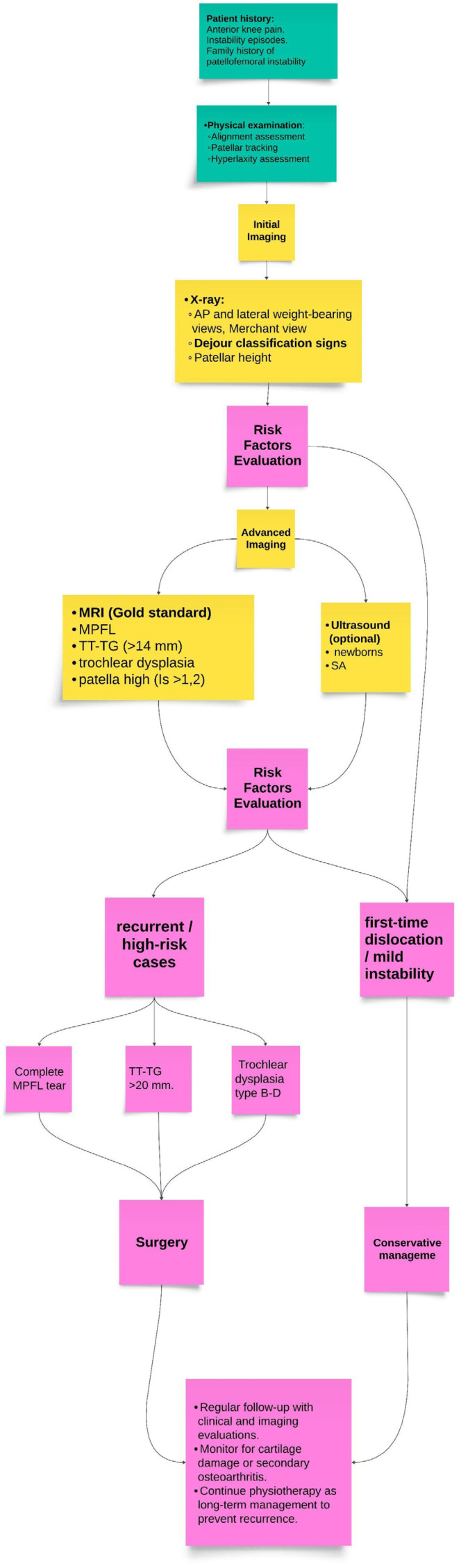
Decision-making framework for the diagnosis of patella instability in children.

Figure 2 presents a decision-making framework for diagnosing PFI in children, emphasizing the integration of imaging modalities with clinical examination. This approach underscores the importance of tailoring diagnostic pathways to individual patient characteristics, such as age, sex, and skeletal maturity, to enhance diagnostic accuracy and effectively guide management strategies. Table 4 represents all patellofemoral morphology risk factors mentioned in included studies and highlight statistically significant factors.

## 4. Discussion

In our work, we addressed the significant yet insufficiently explored issue of PFI in pediatric populations. Despite numerous studies on this topic, there is still a clear lack of definitive guidelines for the diagnosis, clinical decision-making, and management of pediatric patients with PFI. Although MRI remains the primary diagnostic tool, further research is needed to establish more precise diagnostic criteria. Table 2 summarizes how many times each patellofemoral morphology factor was mentioned and how many times it was indicated as a significant risk factor.

We have shown that various risk factors may allow surgeons to evaluate the likelihood of recurrent lateral patellar dislocations (LPD), effectively guiding management strategies. However, the pediatric population presents distinct challenges due to ongoing anatomical changes in the musculoskeletal system as tissues develop. This continuous growth requires a more refined approach, and a single cut-off value for assessment cannot be universally applied. Instead, multiple cut-off values should be considered, factoring in sex, chronological age, and, crucially, biological age. This highlights the need for individualized diagnostic and management protocols to address the complexities of growth and development in pediatric patients.

For example, the measurement of the TT-TG distance is useful in determining appropriate surgical management. In adults, established reference values can be used to assess the need for surgery; however, in the pediatric population, age-based references are necessary during skeletal maturation. Dickens et al. demonstrated that the TT-TG distance varies with chronological age in pediatric patients, suggesting that an age-based approach may be more suitable for skeletally immature individuals with recurrent lateral patellar instability [7]. More studies have further confirmed these findings [10,19]. On the other hand, some studies have reported conflicting findings. For instance, Düppe et al. observed that children had a mean TT-TG distance similar to that of adults, with children experiencing patellar instability showing comparable TT-TG intervals to adults with PFI [14]. Given these inconsistencies, further research is needed. Furthermore, trochlear dysplasia is considered one of the strongest risk factors for PFI [8]. Düppe et al. noted that while several adult-based parameters (such as the CD ratio) serve as effective predictors of patellar instability when observed on pediatric MRI, other parameters, such as the SA, are age-dependent [14]. This implies that SA cut-off values may be needed for the pediatric population.

Authors have also addressed the need for standardization in the face of the observed differences observed between certain measurements from 1.5 T and 3 T MRI scans. For instance, Dickens et al. showed that TT-TG distance measurements were significantly greater at 1.5 T compared to 3 T scans [13]. Similarly, Clinton et al. reported a comparable discrepancy in TT-PCL distance measurements [12]. Although standardization of MRI in this matter is necessary, it remains the primary diagnostic tool for patellar dislocation.

T2 mapping is primarily a research technique and is not routinely incorporated into standard MR imaging protocols in most imaging centers, yet there are some studies in the literature that underline that T2 relaxation time mapping as one of the most widely accepted techniques for cartilage imaging, as it offers valuable biochemical insights and enables the sensitive detection of microstructural changes that occur before observable morphological alterations develop [48,49]. It may be helpful in the diagnosis of cartilage pathologies in young patients with mild symptoms through the early identification of individuals with knee cartilage degeneration [50]. Furthermore, studies have demonstrated that T2 mapping is effective in monitoring disease progression and evaluating treatment efficacy in osteoarthritis [51].

However, it is important to acknowledge that MRI is not the only available diagnostic option. X-ray imaging may serve as an effective first-line assessment, yet results regarding its diagnostic accuracy have been inconsistent. Lippacher et al. demonstrated that radiological indicators such as the crossing sign, supratrochlear spur, bump, and double contour sign could enable clinicians to diagnose PFI without the need for MRI [28]. While this study offers valuable insights, it also has limitations that impact the results. The sample size was relatively small, potentially limiting the study’s broader applicability. Moreover, the methodology lacks details on inter- and intra-observer reliability, raising questions about consistency. The absence of specifics regarding the number of measurements taken further constrains the assessment of the precision of the obtained data. Addressing these methodological issues in future studies would enhance the validity and reliability of the conclusions. Stepanovich et al. also compared MRI and X-ray for the diagnosis of trochlear dysplasia using the Dejour classification. Their findings indicated that MRI-based Dejour had lower inter- and interobserver reliability than radiographic Dejour. On the other hand, Dejour classification on X-Ray is more challenging to measure due to the potential difficulty in obtaining true lateral radiographs, which suggests that other MRI measurements are less subjective and more reproducible [29]. Furthermore, Bernholt et al. have demonstrated that the TT-TG distance can be estimated on X-ray using the Merchant view, showing moderate correlation with MRI TT-TG measurements [27]. These studies collectively underscore the potential role of X-ray imaging in PFI diagnosis, although they also highlight certain limitations.

Finally, while there are a limited number of studies on ultrasonography in the existing literature, it has shown potential for inclusion in the diagnostic pathway for PFI in the pediatric populations. US could serve as a promising screening tool for trochlear dysplasia. In the study by Øye et al., mean and pathological cutoff values for the SA were established, suggesting that screening could extend beyond hip dysplasia to include trochlear dysplasia as well, thus broadening the scope of early diagnostic assessments [37]. The findings of Nietosvaara and Aalto also support the possibility of assessing SA via US [40].

Overall, while MRI and X-ray continue to serve as the primary diagnostic tools for PFI, ultrasonography offers promising complementary approaches. The standardization of imaging protocols and the development of age-specific diagnostic criteria remain crucial for optimizing patient management. Future research should focus on refining these diagnostic tools to enhance accuracy, reliability, and accessibility in pediatric PFI assessment.

## 5. Conclusions

In summary, multiple diagnostic tools are available for the assessment of PFI, each contributing to treatment decisions. However, to enhance the diagnostic accuracy and ensure their appropriate clinical application, further standardization and the development of guidelines are essential, particularly regarding the pediatric population.

## Figures and Tables

**Table 1:** Inclusion and exclusion criteria.

Inclusion Criteria	Exclusion Criteria
Articles written in English.	Articles primarily addressing the treatment of patellar instability with only a brief mention of diagnostic aspects.
Studies specifically focused on identifying and diagnosing patellar discrepancies.	Studies involving patients over 18 years of age.
Prospective and retrospective studies, clinical trials, and case studies concerning patellofemoral disorders.	Non-research articles such as editorials, commentaries, and expert opinions.

**Table 3:** Comparison of imaging techniques.

Imaging Technique	Sensitivity	Specificity	Practical Considerations
MRI	High (85–93%) [41,42]	High for identifying risk factors such as trochlear dysplasia, TT-TG distance, and cartilage damage [43,44].	- Best for soft tissue and cartilage evaluation. - No radiation exposure. - Higher cost and limited availability in some regions.
X-ray	98% for dislocation in lateral view [45]	Moderate (useful for skeletal abnormalities (Dejour classification), dislocation, and patellar height) [46].	- First-line tool for trauma - Identifies osteochondral fractures, patella alta, and Dejour classification.
CT	High (for bone structures)	High (especially for osseous abnormalities).	- Excellent for rotational abnormalities and TT-TG measurement. - Involves radiation exposure; reserved for specific cases.
Ultrasound	Moderate to high	Moderate (depends on operator skill and technique).	- Useful for young children and newborns. - Identifies trochlear dysplasia and sulcus angle. - Radiation-free but operator-dependent

**Table 4:** Patellofemoral morphology risk factors mentioned in included studies (statistically significant).

	MRI	CT	X-Ray	Physical Examination	US
Trochlear dysplasia (trochlear depth, TFA, femoral depth, sulcus depth, trochlear inclination)	22 (20)	2 (1)	3 (3)		4 (4)
Patellar tilt	1 (1)				
TT-TG	12 (12)	1	2 (2)		
TT-PCL	3 (2)				
Tibial tuberosity location	1 (1)				
Patellar high (patella alta, IS, BP, CD)	12 (10)	2 (1)	2		
Patella morphology (Patellar width and length, tendon width, type, Wiberg classifications)	4 (3)	1 (1)			
Lateralization	1				
MPFL	5 (5)				
SA	6 (6)	1 (1)			3 (3)
Alignment	2 (2)				
Cartilage injury	4 (4)				
Dejour classification	2 (1)	1 (1)	3 (2)		
Lateral patellofemoral angle	1 (1)				
VMO	1 (1)				
Gait analysis				2 (1)	
Femoral anteversion	1	1		1	
Tibial torsion		1 (1)			
Fulkerson angle	1 (1)				
Lateral patellar displacement	1 (1)				
Beighton score				1 (1)	

## Abbreviations

Abbreviation	Full name
BP	Blackburne-Peel
CD	Caton Deschamps
CT	Computed tomography
IS	Insall–Salvati
MPFL	Medial patellofemoral ligament
MRI	Magnetic resonance imaging
OA	Osteoarthritis
PFI	Patellofemoral instability
RTG	Radiography
SA	Sulcus angle
TFA	Trochlear facet asymmetry
TT-PCL	Tibial tubercle posterior cruciate ligament
TT-TG	Tibial tubercle trochlear groove
US	Ultrasonography
VMO	Vastus medials oblique
